# 1335. High Serum Levels of Interleukin-10 as a Predictor Factor of In-Hospital Mortality in COVID-19 Patients

**DOI:** 10.1093/ofid/ofab466.1527

**Published:** 2021-12-04

**Authors:** Salome Gomez-Duque, Enrique Gamboa-Silva, Ingrid G Bustos-Moya, Yuli Fuentes, Julian Lozada-Arciniegas, Elsa Daniela Ibañez-Prada, Oriana Narváez - Ramírez, Lina Morales-Cely, Laura A Bravo-Castelo, Paula Ramirez, Daniela Parra-Tanoux, Edar Caceres, Ana Santos, Jhon Londoño, Luis F Reyes

**Affiliations:** 1 Universidad de la Sabana, Chía, Colombia, bogota, Distrito Capital de Bogota, Colombia; 2 Universidad de la Sabana, Cajica, Cundinamarca, Colombia; 3 Universidad de La Sabana, Bogota, Distrito Capital de Bogota, Colombia

## Abstract

**Background:**

Since the spread of SARS-CoV-2 worldwide, there has been the need for scores and biomarkers to identify patients at risk of died or requiring admission to the intensive care units (ICU) admission. Interleukin-10 (IL-10) is released as a response to the infection, stimulating inflammatory pathways in the acute phase response. Thus, previous studies have shown that high serum concentrations IL-10 can be identify patients with severe community acquired pneumonia (CAP). Nevertheless, there is a lack of information regarding the capacity of IL-10 to identify severe COVID-19. Thus, the aim of this study was to determine the capacity of IL-10 as a prediction factor for mortality in hospital admitted patients with COVID-19 compared with CAP patients.

**Methods:**

A prospective observational study was carried out at the Clinica Universidad de La Sabana, Colombia. Patients older than 18 years and old, hospitalized due to COVID 19 or CAP, were included. Patients were stratified into COVID-19 and non-COVID-19 patients. IL-10 levels were quantified in serum samples using the LUMINEX technology. Serum samples were collected within the first 24 hours of hospital admission. Afterward, concentrations of interleukinwere statistically compared among groups. ROC curves were calculated.

**Results:**

A total of 88 patients with CAP and 152 patients with COVID-19 were enrolled in the study. The median [with IQR] serum concentration of IL-10 were higher in those patients who died (81.1 [30.7-148.9] vs 18.8 [8.3-48.4] p-value < 0.0001). Then, comparing the study group, the median concentration of IL-10 levels among patients deceased by COVID-19 were higher than patients those who survived (85.1 [40-149.8] vs 32.4 [13.9-56.7] p-value < 0.001). In addition, IL-10 levels were higher in patients who survived COVID-19 compared with those who survived CAP (32.4 [13.9-56.7] vs 10.6 [4.9-18] p-value < 0.0001). The area under curve (AUC) ROC of IL-10 to predict mortality risk was 0.754 for all cohort. DeLong′s test comparing ROC curves in COVID-19 and CAP patients had a *p= 0.744.*

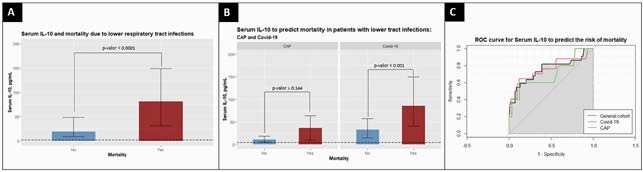

**Conclusion:**

High serum levels of IL-10 are a good predictor of in-hospital mortality among COVID-19 patients. However, this risk association was not observed in CAP patients. Further studies are needed.

**Disclosures:**

**All Authors**: No reported disclosures

